# Hillslope Processes Affect Vessel Lumen Area and Tree Dimensions

**DOI:** 10.3389/fpls.2021.778802

**Published:** 2021-12-03

**Authors:** Jakub Kašpar, Pavel Šamonil, Martin Krůček, Ivana Vašíčková, Pavel Daněk

**Affiliations:** ^1^Department of Forest Ecology, The Silva Tarouca Research Institute, Brno, Czechia; ^2^Department of Forest Botany, Dendrology and Geobiocoenology, Faculty of Forestry and Wood Technology, Mendel University in Brno, Brno, Czechia; ^3^Department of Botany and Zoology, Faculty of Science, Masaryk University, Brno, Czechia

**Keywords:** stem eccentricity, height limitation, hillslope processes, tree stability, wood anatomy, biogenic creep, *Quercus*, *Fraxinus*

## Abstract

The height growth of the trees depends on sufficient mechanical support given by the stem and an effective hydraulic system. On unstable slopes, tree growth is affected by soil pressure from above and potential soil erosion from below the position of tree. The necessary stabilization is then provided by the production of mechanically stronger wood of reduced hydraulic conductivity. Unfortunately, the interaction between tree growth (both radial and axial) and stabilization in the soil is still insufficiently understood. Therefore, in this study, we aimed to quantify the impact of hillslope dynamics on the degree of tree growth and hydraulic limitation, and the potential effect on tree height growth and growth plasticity. To evaluate this effect, we took four cores from 80 individuals of *Quercus robur* and *Fraxinus excelsior* and measured tree-ring widths (TRWs) and vessel lumen areas (VLAs). The tree heights were evaluated using a terrestrial laser scanner, and local soil depth was measured by a soil auger. Our data showed a significant limitation of the tree hydraulic system related with the formation of eccentric tree-rings. The stem eccentricity decreased with increasing stem diameter, but at the same time, the negative effect of stem eccentricity on conduit size increased with the increasing stem diameter. Even though this anatomical adaptation associated with the effect of stem eccentricity differed between the tree species (mainly in the different degree of limitations in conduit size), the trees showed an increase in the proportion of hydraulically inactive wood elements and a lowered effectiveness of their hydraulic system. In addition, we observed a larger negative effect of stem eccentricity on VLA in *Quercus*. We conclude that the stabilization of a tree in unstable soil is accompanied by an inability to create sufficiently effective hydraulic system, resulting in severe height-growth limitation. This affects the accumulation of aboveground biomass and carbon sequestration.

## Introduction

Trees drive ecosystem dynamics in many terrestrial ecosystems and are considered as ecosystem engineers (Pan et al., 2011; Jones, 2012; Jerin and Phillips, 2020). Through their size, the trees affect the distribution of light, nutrients, water, and the microclimate. Tree growth is mainly controlled by the climate (Rossi et al., 2016) and local disturbances (e.g., Frelich, 2002). However, on unstable slopes, the growth of trees may be significantly disturbed by the hillslope processes (e.g., Šilhán, 2017, 2019; Tumajer and Treml, 2019; Kašpar et al., 2020). Frequent stem tilting leads to a continuous restoration of the stem position toward vertical (Harker, 1996; Šilhán, 2015). In such cases, the phytohormonal concentrations within the tree are imbalanced (Aloni, 2007), which results in eccentric growth and the production of so-called reaction wood (Pallardy, 2008; Bräuning et al., 2016).

The size of the conduit elements of trees increases ontogenetically with the increasing distance from the stem base to the apex (Olson et al., 2014; Kašpar et al., 2019; Fajardo et al., 2020). This general pattern is partly influenced by climate (e.g., Fonti et al., 2013; Castagneri et al., 2017; Jevšenak et al., 2018a,b) and partly by the formation of reaction wood (Jourez et al., 2001). This reaction wood generally has vessels with smaller lumens (Tumajer et al., 2015) and different density (Heinrich et al., 2007). The result is wood with higher mechanical strength, but substantially lower hydraulic conductivity (Pallardy, 2008; Ruelle, 2014; Bräuning et al., 2016; Tumajer and Treml, 2019). The long-term compensation of external pressures (and related formation of reaction wood) may thus potentially lead to a significant limitation of axial growth, due to the inability to create a sufficiently efficient hydraulic structure. Therefore, the actual height reached of the affected trees given by the environmental conditions may be considerably lower than the potential height produced by unlimited growth (Ryan and Yoder, 1997; Ryan et al., 2006). Additionally, limitation of the axial dimensions limits the increase in aboveground biomass allocation. Along with the fact that the production of smaller vessels requires less carbon (Koçillari et al., 2021), the occurrence of hillslope processes may limit the abilities of trees to sequester carbon.

*Quercus robur* is among the most common lowland tree species in Europe, and in natural forests, it is usually accompanied by *Fraxinus excelsior* (San-Miguel-Ayanz et al., 2016). Both the tree species are ring-porous woods and thus have in general a similar anatomical structure. However, their anatomy slightly differs in some significant aspects, such as the mean vessel size, vessel density, and tendency of vessels to grouping to cluster (Ruffinatto and Crivellaro, 2019). As ongoing climate change also affects the intensity and structure of slope processes, the species-specific responses to the hillslope dynamics may influence the competitive ability of tree species in an ecosystem. In the chain of links, all of this can also be reflected in the species composition of forest ecosystems and potentially affect between-tree species competition.

In our study, we focused on these two tree species growing at a site significantly affected by soil and regolith creep. The instability caused by soil creep should be reflected in the production of reaction wood over a long time horizon, with this stabilization effect of trees in soil interacting with the significant hydraulic limitation. We hypothesized that this interaction may potentially limit the height growth of trees and consequently the aboveground biomass allocation.

## Materials and Methods

### Study Site

**FIGURE 1:**
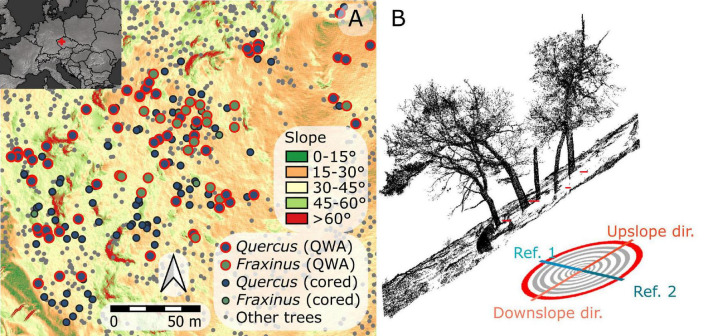
Map of the study site **(A)** and schematic image of field data sampling **(B)**.

The research was conducted in the Velká Pleš Reserve, protected since 1984. The site is located in the central part of Czechia (49.99°N, 13.81°E; Figure 1A), Czechia, at an altitudinal range from 347 to 496 m a.s.l. (Figure 1A). The annual temperature is 8.2°C and annual precipitation 574 mm (Tolasz et al., 2007). Cambisols, Leptosols, or tuff outcrops occupy gentle and steep slopes of average inclination approximately 23 degrees. The dominant tree species at this site are *Quercus robur* and *Fraxinus excelsior*, supplemented by *Tilia cordata* and *Carpinus betulus*.

Within the reserve, we selected a smaller subplot with visible signs of hillslope processes (Pawlik and Šamonil, 2018); as shown in Figure 1B and Supplementary Figure 1, for examples, of selected trees. The area of the subplot was 2 ha (100 m × 200 m) and average slope steepness in this part was 27 degrees, ranging from 0 to 45 degrees on terrain lacking rock outcroppings.

### Field Measurements, Sampling, and Sample Preparation

A tree census was conducted at the study site in 2012. The exact positions (precision of ca. 1 m) of all the standing and lying trees of diameter at breast height (DBH) >7.5 cm were recorded, together with additional attributes (tree species, DBH, and health status). These data served us as the starting information for the creation of a stem-position map that included the basic characteristics.

The subplot was covered by terrestrial laser scanning (TLS) in 2019 using a Leica P20 with 22 scanner placements. These data were used to refine the tree map, to create a precise digital model of relief (DMR) of the subplot, and a precise model of each tree. Based on the DMR, the slope of the each stem base was calculated in detail. The distance from the stem base to the apex (stem length) was determined from the tree models.

In the close vicinity of 118 selected tree individuals, soil depth was evaluated according to Shouse and Phillips (2016). We applied five soil augers within a radius of 1 m around the tree and recorded the depth to the point of refusal. To avoid distortions that may be related to the effect of isolated stones in soils, we considered the deepest record to be the soil depth for that particular tree.

In total, we collected core series from 80 standing non-rotten trees for the dendrochronological analysis (57 of *Quercus* and 23 *Fraxinus*). All the samples were taken using an increment Pressler’s borer. From each tree, a total of four cores were taken, one downslope, one upslope, and two in contour directions (Figure 1B; as shown in ref Kašpar et al., 2020), at approximately 50 cm above the surface (measured from the contour). Each sample was visually checked and only samples without rot and within an estimated distance from the pith lower than 3 cm (Applequist, 1958) were accepted for further analysis.

The collected samples were dried at room temperature and fixed into wooden slices. The prepared cores were then soaked in water for a couple of hours, and then the surfaces of all cores were cut using a WSL microtome (Gärtner and Nievergelt, 2010). Subsequently, all cores were scanned using an Epson LA2400 high resolution scanner (Epson, Japan) at 1200 DPI resolution and measured in WinDENDRO software. Cross-dating of measured tree-ring series was done in PAST5 software with the simultaneous use of COFECHA software (Grissino-Mayer, 2001).

The trees with measured soil depth were selected for the quantitative wood anatomy analysis (QWA). In these samples, we enhanced the contrast between the tree-ring wood mass and vessel lumens applying a black ink marker and white chalk (Gärtner and Schweingruber, 2013). These samples were again scanned in high resolution (3200 DPI). To obtain the best contrast between black (wood mass) and white (vessel lumina), these images were improved using image manipulation software (GIMP^©^). The samples were measured using ROXAS^©^ software (von Arx and Carrer, 2014), resulting in data on the ring areas and vessel lumen areas (VLAs) of all the visible vessels in all the visible tree-rings.

In the end, we had two types of synchronized time series: a tree-ring width (TRW) dataset and a dataset of VLAs.

### Data Analysis

For each year, we calculated the stem diameter based on TRW and estimated the distances of each core to the pith. For each tree, mean TRW series were calculated (using all four cores). TRW series were then detrended in R (R Core Team, 2021) using the dplR package (Bunn, 2008) and using a negative exponential curve (Rybníček et al., 2016) to eliminate the influence of stem diameter on radial growth. Then, *via* detrending, we obtained: (1) indexed TRWs for a mean growth curve of each tree; and (2) indexed TRWs for each core from an individual tree (TRW index of each core was calculated using a negative exponential obtained by detrending the mean TRW curve of the tree).

To quantify the eccentricity of radial stem growth, we calculated a variation coefficient from the widths of corresponding tree-rings (TRW variation). We chose this approach due to its dimensionless and because slow hillslope processes (such as creep) may be manifested in different parts of the stem with the changing tree size (Kašpar et al., 2020); as shown in Supplementary Figure 1.

Data measured by ROXAS were precisely cross-dated with the measured TRW series. Then, each measured vessel was identified with the corresponding calendar year and direction in the given tree. Based on these data, we calculated the mean VLA (VLA_mean_) and areas of the largest vessels represented by the 90^th^ percentile of VLA (VLA_90_). Mean series of cell lumen areas of each direction within the trees were also calculated. For each cell we calculated its theoretical hydraulic conductivity (Kh), which reflects the Hagen-Poiseuille law regarding increasing efficiency in water transport with increasing vessel size (Tyree and Zimmermann, 2002). While calculating Kh, we assumed an ideal circular section of each evaluated vessel (Gebauer and Volařík, 2013), following the equation:


$$Kh=\frac{\rho A^{2}}{8\eta \pi }$$

where Kh is theoretical hydraulic conductivity of a given vessel (kg m s^–1^MPa^–1^), ρ is water density at 20°C (998.205 kg m^3^), A is the lumen area of a given vessel (m^2^), and η is the viscosity of water at 20°C (1.002.10^–9^MPa s^–1^).

Specific hydraulic conductivity (Ks) for each tree-ring was then calculated from Kh following the equation:


$$Ks=\frac{\sum_{i=1}^{n}Kh_{i}}{A_{xyl}}$$

where Ks is the specific hydraulic conductivity of the tree-ring xylem (kg m^–1^ s^–1^MPa^–1^), and A_*xyl*_ is the measured area of the tree-ring (Tyree and Zimmermann, 2002). This was done separately for each tree-ring in a given direction (equation above), as well as for the combination of the annual ring in all the evaluated directions. For this purpose, the sum of Kh in all four directions together with measured areas of each tree-ring was used. Ks then represents a theoretical value of the hydraulic conductivity (Tyree and Zimmermann, 2002). This calculation does not consider vessel axial length (Jacobsen et al., 2012) or the effect of vessel grouping (von Arx et al., 2013). The missing tree-rings in individual cores had a Ks value of 0 in our dataset.

### Statistical Analysis

Differences in the basic characteristics (Table 1) between the species were evaluated by using ANOVA. To model TRW variation, VLA_mean_, VLA_90_, and Ks, we used linear mixed effect models (nlme package in R; Pinheiro et al., 2019; R Core Team, 2021).

**TABLE 1:** Basic characteristics of the analyzed trees.

	Number of trees	Tree age	Tree height (m)	Stem diameter (cm)	Slope (°)	Soil depth (cm)	Cross section var.	VLA_mean_ (μm^2^/1000)	VLA_90_ (μm^2^ /1000)	Specific hydraulic conductivity (kg.m^–2^.MPa^–1^.s^–1^)
All data	80	182	11.65	28.1	27.9	64	0.14	16372	35609	56.05
		± 22	± 2.86	± 7.6	± 6.0	± 21	± 0.07	± 3540	± 8743	± 28.56
*Quercus*	57	183	11.37	27.7	27.9	68	0.14	17180	38317	59.98
		± 15	± 3.11	± 7.6	± 6.6	± 21	± 0.06	± 3637	± 8271	± 30.34
*Fraxinus*	23	178	12.36	28.9	28.1	55	0.17	14371	28901	53.77
		± 33	± 2.04	± 7.9	± 4.4	± 20	± 0.07	± 2341	± 5867	± 23.83
Difference						*	*	**	***	.

Statistical significances of differences between species are marked by a dot (p < 0.1), * (p < 0.05), ** (p < 0.01), or *** (p < 0.001).

Fixed effects used to model TRW variation were the species (*Quercus*/*Fraxinus*), stem diameter (cumulative diameter at 50 cm above stem base), TRW index (residuals obtained by the detrending procedure), slope steepness, soil depth, and direction with the widest tree-ring in a particular year (as shown in the R code of the model in Supplementary Method 1).

The evaluations of VLA_mean_, VLA_90_, and Ks were done first for data of corresponding years averaged over all directions. For all the models, we used the same predictors: tree species, stem diameter, TRW variation (stem eccentricity), TRW index, slope steepness, and soil depth (as shown in the R code of the model in Supplementary Method 2). Finally, we modeled non-averaged VLA_mean_ and Ks values from all directions using the same predictors as for models, evaluating the means supplemented by the core direction (as shown in the R code of the model in Supplementary Method 3).

The stem diameter and TRW index were included in the models as degree 2 orthogonal polynomials. The response variables VLA_mean_ and VLA_90_ and the explanatory variable stem diameter were log transformed. The values of soil depth and slope steepness were standardized. In all the models, tree ID was used as a random effect and temporal autocorrelation was accounted for using the autoregressive moving average (ARMA) autocorrelation structure with parameters set to minimize model AIC (as shown in Supplementary Methods 1–3; Ives et al., 2010).

In all the cases, we first created a full model with all the possible interactions, which was subsequently simplified by excluding insignificant (*p* > 0.05) interactions. However, when modeling VLA_mean_, VLA_90_, and Ks, insignificant interactions were kept in the model if they were significant in another model. This was done to keep the models mutually consistent. The explanatory power of models was assessed by means of conditional and marginal R_2_ using the r.squaredGLMM function from the MuMIn package (Nakagawa and Schielzeth, 2013; Bartoń, 2016).

The exponent of the power relationship between the vessel-related properties (VLA_mean_, VLA_90_, and Ks) and stem diameter was fitted using the linear regression on log-log transformed data up to a stem diameter of 20 cm. In the case of VLA_mean_ and VLA_90_, we also fitted a relationship with a fixed linear coefficient of 0.4, which corresponds to the theoretical universal power relationship between cell diameter and tree height (Tyree and Zimmermann, 2002; Kašpar et al., 2019). In addition, we used the relationship between vessel dimensions and stem diameter as reported by Klesse et al. (2020). Using this approach, it was possible to evaluate the potential for height limitation in the analyzed trees.

## Results

In total, we analyzed data of 15,353 tree-rings in 80 trees (57 *Quercus* and 23 *Fraxinus*). In general, individuals of both the tree species were the same average age and size and grew at slopes of similar inclination (Table 1). On the other hand, significant differences (*p* < 0.05) between the species were found in soil depth, stem eccentricity, and wood anatomy (Table 1). *Quercus* created significantly (*p* < 0.05) larger cells (Table 1). Both the tree species did not show significantly (*p* < 0.05) different Ks (Table 1).

### Stem Eccentricity and Tree Ontogenesis

**FIGURE 2:**
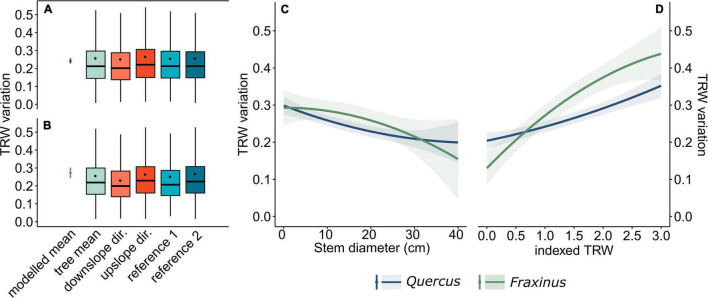
Modeled and observed stem diameter variation of *Quercus* **(A)** and *Fraxinus* **(B)**, the modeled effect of stem diameter on tree-ring width (TRW) variation **(C)** and the modeled effect of indexed TRW on TRW variation **(D)**. The boxplots represent 25th and 75th percentiles, thick horizontal lines represent medians and dots represent the mean values. Whiskers denote the 1.58*inter-quartile range (ggplot2), while outliers are not shown.

Most of the TRW variations were caused by the presence of the widest tree-rings in the upslope direction (32% cases). The correlation of stem eccentricity and TRW variation for this direction was significantly higher (*p* < 0.001) than for other evaluated directions (Figures 2A,B). The result of the linear mixed effect model revealed that overall TRW variation significantly (*p* < 0.05) decreases with the increasing stem diameter (Figure 2C), with a statistically insignificant difference between the two species. According to the model, the variation in TRWs was significantly greater in years with high radial growth (Figure 2D) and was significantly higher in the case of *Fraxinus* (*p* < 0.05). Additionally, the model showed the highest range of predicted TRW variation values for the stems of sizes from ∼5 to 30 cm, due to the high indexed TRW (Supplementary Figure 2).

### The Influence of Stem Eccentricity on Tree-Hydraulic Performance

**FIGURE 3:**
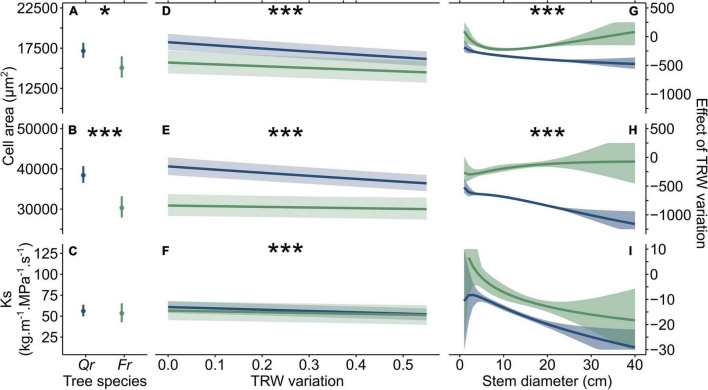
The modeled mean values of mean vessel lumen area (VLA_mean_), 90th percentile of VLA (VLA_90_), and specific hydraulic conductivity (Ks) (**A–C**, respectively) and the effect of TRW variation on VLA_mean_, VLA_90_, and Ks of fitted models: (**D–F**, respectively), and changes in the slope of the linear regression of TRW variation with increasing stem diameter in relation with VLA_mean_, VLA_90_, and Ks (**G–I**, respectively). Blue color represents *Quercus* and green color represents *Fraxinus*. Statistical significance of differences in the trends between species is expressed by asterisks (* is *p* < 0.05 and *** is *p* < 0.001).

Increasing TRW variation negatively affected all the studied anatomical features (Figures 3D–F), and in the case of VLA_mean_ and VLA_90_ affected both the species in the same way but with different degrees of expression. Increasing TRW variation of about 0.1 caused a decrease of VLA_mean_ by ∼364 and ∼214 μm^2^, and of VLA_90_ by ∼752 and ∼129 μm^2^ in *Quercus* and *Fraxinus*, respectively. Considering the mean modeled values of VLA_mean_ and VLA_90_, VLA_mean_ was lower by about ∼2.1, ∼1.4% and VLA_90_ by ∼2.0, ∼0.4% (*Quercus* and *Fraxinus*, respectively). The Ks values decreased to ∼1.6 and ∼1.0 kg.m^–1^.s^–1^.MPa^–1^ in *Fraxinus* and *Quercus*, respectively, compared with the modeled mean values of about ∼0.95 and ∼0.56%. The negative effect of TRW variation on both the VLA_mean_ and VLA_90_ of *Quercus* increased with the increasing stem diameter, with a much stronger effect in VLA_90_ than in VLA_mean_. On the contrary, the effect on vessels of *Fraxinus* remained constant (Figures 3C,F). On the other hand, the strength of the negative effect of TRW variation on Ks increase with the increasing stem size (consistently for both the species; Figures 3G–I). This indicates different anatomical adaptations for the two studied tree species.

Considering the effect of other predictors, VLA_mean_, VLA_90_, and Ks increased with the increasing stem size (Supplementary Figures 4A–C). The effect of indexed TRW on VLA_mean_, VLA_90_, and Ks was generally negative. However, the largest vessels (VLA_90_) were created when indexed TRW was from ∼1 to ∼2 (Supplementary Figures 4D–F).

Soil depth and slope separately had insignificant effects on VLA_mean_, VLA_90_, and Ks. However, both the predictors became significant in the interactions with stem diameter (in the case of slope) or with indexed TRW (in the case of soil depth). The effect of slope on VLA_mean_, VLA_90_, and Ks was negative considering trees of stem diameter from 10 to 25 cm growing on steeper slopes (Supplementary Figures 4G–I). This trend was significant (*p* < 0.001) for VLA_mean_ and VLA_90_. The effect of soil depth was positive considering narrow tree rings and gradually decreased with the increasing annual growth (Supplementary Figures 4J–L).

### The Effect of Stem Eccentricity on Tree Height

The analysis of the effect of TRW variation on VLA_mean_ and Ks in particular directions showed consistent results with averaged values per entire stems (Supplementary Figure 5). The trees of both the species created significantly smaller vessels in the upslope direction (*p* < 0.05), and thus wood with lower Ks (*p* < 0.05). However, the effect of TRW variation in VLA_mean_ and Ks did not differ among the studied directions (Supplementary Figure 5).

**FIGURE 4:**
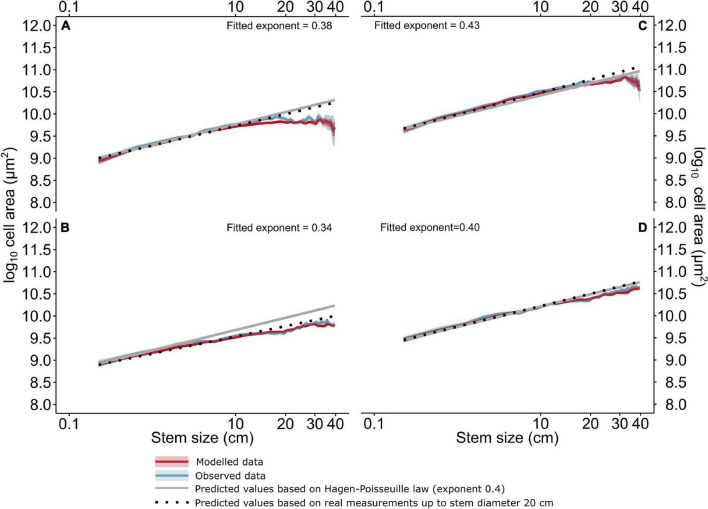
The bootstrapped measured and predicted values of VLA_mean_ **(A,C)**, VLA_90_ **(B,D)** and differences from the expected values based on a linear regression of log-log transformed measured data. The solid black lines represent predicted values based on the Hagen-Poisseuille law, the dotted black lines show predicted values based on real measurements up to the stem diameter 20 cm. The solid red lines represent values predicted by the linear mixed effect models, and the solid blue lines represent measured values, respectively, their difference from predicted values based on the Hagen-Poisseuille law. *CI*s represent the bootstrapped means (*p* = 0.95) of both observed and modeled data for stem size intervals of 1 cm.

Stem size was a statistically significant predictor (*p* < 0.001) of VLA_mean_, VLA_90_, and Ks. The linear exponent of VLA_mean_ widening of trees of stem diameter <20 cm was 0.38 (*Quercus*) and 0.34 (*Fraxinus*), respectively (Figures 4A,C). Considering VLA_90_, the exponent was close to 0.4 (Figures 4C,D). The exponent of the basipetal widening of trees of the stem size >20 cm was significantly lower, ∼0.14 for VLA_mean_ and from 0.21 (*Fraxinus*) to 0.32 (*Quercus*) considering VLA_90_. The comparison with the observed and modeled values of VLA_mean_ showed a negative deviation of VLA_mean_ and VLA_90_ from the trends of basipetal widening in both the tree species (Figure 4). The difference of VLA_mean_ was clear at stem diameters higher than 5–15 cm (*Fraxinus*) and 10–15 cm (*Quercus*). In contrast, the difference in VLA_90_ was observed at stem dimensions >15 cm in *Fraxinus* and >20 cm in *Quercus*. Similar thresholds were observed for Ks (not shown).

## Discussion

In Velká Pleš, we observed an increasing limitation of tree conduits due to stem eccentricity, and which increased with the increasing stem size. The growth of trees on unstable slopes is associated with frequent stem tilting and the related production of mechanically stronger wood (Jourez et al., 2001; Bräuning et al., 2016). Simultaneously, the trees tend to stabilize soils on slopes that has been found to reduce the stem eccentricity of larger trees (Di Iorio et al., 2005; Šilhán, 2019). The long-lasting production of reaction wood may, however, result in the tree height limitations and potentially reduce biomass allocation.

### The Influence of Tree-Ring Width Variation and Stem Diameter on Vessel Lumen Area

As a reaction to external pressures, the trees in our study produced eccentric tree rings composed of wood with reduced hydraulic conductivity (Figure 3), confirming the results of previous studies (e.g., Heinrich and Gärtner, 2008; Tumajer et al., 2015; Tumajer and Treml, 2019; Piermattei et al., 2020). The linear mixed effect models performed in this study revealed a higher decrease in both the VLA_mean_ and VLA_90_, of *Quercus* and *Fraxinus*, respectively (Ballesteros et al., 2010). An increase in the variation in TRW by 0.1 caused a significant decrease in VLA. Considering the Hagen-Poisseuille law, the mean reduction in theoretical hydraulic conductivity (Kh) of a mean vessel was ∼4.2% (*Quercus*) and ∼2.8 % (*Fraxinus*), which is two times higher than the relative decrease in VLA_mean_. The limitation of Kh of the largest vessels was lower (∼3.9 and ∼0.9%), however, suggesting the importance of the largest (earlywood) vessels in maintaining the hydraulic stability of ring-porous tree species, similarly as the earlywood cells in the reaction wood of conifers (Domec and Gärtner, 2003). In another study, a decrease in VLA was only partly compensated for by the production of a larger number of smaller vessels, but this effect was not sufficient, and the increase in TRW variation had a negative effect on Ks (Tumajer and Treml, 2019).

Even though both the studied tree species showed a reduction in the hydraulic efficiency of the wood produced, the degree of expression differed between the studied tree species. Both the tree species showed a different degree in the decrease of VLA due to stem eccentricity, while Ks remained similar. The limitation of VLA size due to stem eccentricity was more significant in *Quercus* (because of the production of generally larger vessels; Ballesteros et al., 2010). On the other hand, *Fraxinus* created more wood without vessels (wood with minimal hydraulic conductivity). Therefore, generally more eccentric *Quercus* individuals were significantly smaller than the narrower ones. This, however, did not apply for the *Fraxinus* individuals, most likely because of only slight decrease of Kh. All this suggests a higher growth plasticity of *Fraxinus* (compared with *Quercus*) and its higher suitability for growth on steep slopes.

As shown by our data (Figure 4), the exponent of the basipetal widening increased with the increasing stem diameter up to 20 cm. Since tree height is related to the stem diameter (Bontemps et al., 2010), the effect of stem diameter on the basipetal widening should, to some extent, be of similar character as the well-documented effect of tree height (Anfodillo et al., 2006, 2013; Olson et al., 2014, 2021; Kašpar et al., 2019; Fajardo et al., 2020). The ideal coefficients of basipetal widening, following the Hagen-Poisseuille law, are 0.2 for cell diameter (Anfodillo et al., 2006, 2013; von Arx and Carrer, 2014; Olson et al., 2021) and 0.4 for conduit area (Tyree and Zimmermann, 2002; Kašpar et al., 2019; Fajardo et al., 2020). Nevertheless, in Velka Ples, this relationship was true only up to a stem diameter of ∼15 cm. When considering the sizes of higher dimensions (>15 cm), the exponent was significantly lower 0.1–0.16. On the other hand, the relationship among stem diameter, the VLA_90_, and Ks remained unchanged up to ∼15 cm. The decrease in vessel size due to the stem eccentricity in larger trees was partially compensated for by a higher number of smaller vessels in a larger assimilation area (Tumajer and Treml, 2019). Anyway, our results revealed a linear relation of stem diameter and tree height in relatively thin and young trees but not larger trees (stem diameters larger than ∼15 cm). Contrarily, such a change in trend was not observed in the relationship of tree age and DBH (Supplementary Figure 7).

The strongest predictor of VLA size is pathway length, explaining more than 63% of the data variability, while climate factors explain up to ∼6% of the variability (Rosell et al., 2017). The effect of TRW explained in our study was ∼2.5, with the effect being negative. In contrast to the TRW variance, however, the effect of climate may be both positive and negative. In any case, the negative effect of TRW variation on conduit size higher than 5% was observed in 28.4% of the cases. This indicates a high degree of VLA limitation due to the hillslope processes and a comparable effect of TRW variance by climate. This is especially true when considering that the effect of stem tilting persists for several growing seasons (Šilhán and Stoffel, 2015). In contrast, the climate affects VLA in a narrower period, usually in the current or following growing season (Castagneri et al., 2017).

When analyzing the decrease in vessel size due to TRW variation in different directions, the smallest vessels were observed in the upslope direction. Similarly, the lowest values of Ks were observed in the upslope direction but also in the downslope direction. This is in agreement with the presumed occurrence of tension wood in deciduous trees (Heinrich et al., 2007). At the same time, differences in the decrease of the VLAs and specific hydraulic conductivity among all the directions have been found to be insignificant (Tumajer and Treml, 2019). Therefore, a constant decrease in hydraulic parameters across the stem appears to be a more robust proxy than a comparison of TRWs (Supplementary Figure 5). This is especially true when considering the complexity of biomechanical interactions of trees in soil (mainly of smaller trees), which can force the eccentric growth in other directions than would be generally expected (Wistuba et al., 2015; Malik et al., 2016; Kašpar et al., 2020).

### Changes in the Tree-Ring Width Variation Due to Increasing Tree Dimensions

The TRW variation of trees in our study gradually decreased with the increasing stem diameter, assuming the stabilization effect of trees in soil (Šilhán, 2019; Kašpar et al., 2020). It is commonly accepted that enhanced tree stability is connected with an increasing root biomass penetrating the deeper soil horizons (e.g., Di Iorio et al., 2005; Šilhán, 2017), which can be increased by the tree weight. On the other hand, hard fixation and increasing tree height (manifesting in increasing leverage) may cause compensation for tree tilting more difficult. This assumption is supported by our results showing the increasing negative effect of TRW variation with increasing stem diameter observed on both VLA and Ks (Figure 3).

Both modeled and measured data showed the decreasing trends in TRW variation with the increasing stem diameter, with the highest values of TRW variance recorded for stems of ∼5–20 cm in DBH (data not shown). Similar results were reported by Kašpar et al. (2020), who observed a gradual decrease in stem eccentricity with increasing stem size, and Šilhán (2019), reporting the highest susceptibility to stem eccentricity in trees from 30 to 60 years of age (corresponding with individuals of stem size ∼5–15 cm). On the contrary, Šilhán and Stoffel (2015) found *F. sylvatica* individuals of DBH = 33 cm to be the best indicators of landslide activity. A comparison of both the studies with our results suggests that threshold when the trees start to be susceptible to the mass movements or become a stabilizing factor on slopes is not universal, and probably depends on many aspects, such as soil texture, geology, amount of rock fragments, precipitation, and slope steepness. Additional research in different regions and environmental conditions would be valuable.

### Height Limitation Due to Hillslope Processes

Our results demonstrate growth plasticity moderated by soil pressure. Under such pressure, *Quercus* starts to form vessels of significantly smaller sizes and increased density (Gärtner et al., 2003). On the contrary, the *Fraxinus* vessel sizes are less reduced in such conditions, but more wood without vessels is created (Tumajer and Treml, 2019). The tree species thus show a different adaptation in their anatomical structure to external pressures. Although the response to soil pressure varies between the tree species, adaptations in both the cases result in the formation of wood with higher mechanical strength and reduced specific hydraulic conductivity. On the other hand, the loss of several smaller cells causes a significantly lower reduction of hydraulic conductivity than the loss of the same amount of larger cells. Production of smaller cells may then reduce the vulnerability of trees to cavitation (Gullo et al., 1995).

The feedback between tree growth and gradual slope stabilization found in our results suggests that the stabilizing effect of trees is accompanied by a severe reduction in the ability of trees to create an effective water transport system. Despite the fact that the hydraulic architecture of the tree has a large overcapacity (Dietrich et al., 2018), this likely results in their axial limitation (Koch et al., 2004), as supported by several results. First, there is evidence of a nonlinear relationship between the stem diameter and VLA (Figure 4). Unlike Domec and Gärtner (2003), our data confirmed a similar relationship of basipetal vessel widening due to increasing stem diameter as due to increasing tree height (Anfodillo et al., 2013; von Arx and Carrer, 2014; Klesse et al., 2020). However, this relationship was relevant up to the 20 cm, while deviance was observed already from 5 and 15 cm in *Fraxinus* and *Quercus*, respectively. For a stem diameter larger than 20 cm, the coefficient of basipetal widening considerably decreased under 0.15. This causes a loss of hydraulic capacity that is likely to be more than tree is able to replace after one disturbance event (Dietrich et al., 2018), especially considering the long-term effect of hillslope processes on the tree stems. The second evidence is a comparison of the random effects of the model of stem eccentricity. The stem length of more eccentric trees gradually decreased by 1.5 m, while the random effect of TRW variation increased by about 0.1 (*p* > 0.05). The insignificance of this result may be explained by the fact that all trees in our area were affected by the hillslope processes. In addition, the stem diameter was not significantly correlated with tree height. Additionally, the observed decrease in the coefficient of vessel widening appeared in the same stem diameters as the highest variance in the susceptibility of trees to eccentricity (Figure 4 and Supplementary Figure 2). Finally, there was a change in the effect of the slope inclination for the stems of dimensions wider than 5 cm (Supplementary Figure 4).

Limited height growth goes hand in hand with the limitations in aboveground biomass allocation (e.g., Chojnacky et al., 2014). The production of larger vessels requires a significantly higher amount of carbon (Koçillari et al., 2021). Therefore, a significant reduction in vessel basipetal widening and the associated height growth limitation thus inevitably leads to a reduction in the ability of trees to act as carbon sinks. This limitation gradually increases with the increasing stem size.

### Model Parameterization and Metrics Used

In this study, we avoided evaluating stem eccentricity using previously applied approaches based on the comparisons of the widths of opposite tree rings (e.g., Tumajer and Treml, 2013). Indeed, we used the coefficient of variation in TRWs, mainly because of the effect of biomechanical interactions or hillslope processes may potentially cause eccentricity not only in one (expected-upslope) direction but alternately in different multiple directions or potentially in the opposite direction than expected (Wilson and Gärtner, 2011; Wistuba et al., 2015; Malik et al., 2016; Kašpar et al., 2020). Moreover, the coefficient of variation is a dimensionless metric, which is useful when studying stem eccentricity in multiple directions and comparing the eccentricity of trees of different sizes. The significantly (*p* < 0.05) higher TRW variation in our results, caused by the wide tree rings in the upslope direction, was observed only when comparing with the downslope and reference direction 1 (Figure 2). The widest tree-rings were observed in the upslope direction only in 36% of the cases; therefore, the observed eccentricity in other directions demonstrates both the complexity of hillslope and biomechanical processes (Šamonil et al., 2018) in the shaping of stem morphology (Kašpar et al., 2020).

The slope and soil depth alone were insignificant in the linear mixed effect models. However, both the predictors became statistically significant in the interactions with stem diameter and indexed TRW. Increased tree weight or a higher soil mass pushing on the root system increases the external pressure, and leads to a further intensification of the negative effects of slope and soil depth on the TRW variation or the hydraulic system of the tree (both VLA and Ks).

In our study, we did not evaluate the influence of climate metrics on VLAs or specific hydraulic conductivity. Even though many studies have reported tree height as a dominant factor of basipetal widening (Fajardo et al., 2020), some recent studies have highlighted the influence of temperature (Jevšenak et al., 2018a) and precipitation (Castagneri et al., 2017; Jevšenak et al., 2018b) on VLAs and hydraulic conductivity. In addition, climate has an undeniable influence on the tree ring widths of both the studied tree species (Rybníček et al., 2016; Roibu et al., 2020), and specifically influences the growth of trees of different dimensions (Trouillier et al., 2019). To maximally eliminate the influence of climate, our study was based on similarly large and aged trees (Table 1) from one site (Figure 1). Nevertheless, we are aware that part of the variability associated with the tree dimensions, climate, and possibly age, remained unexplained by our models. However, the addition of climate would not have allowed us to fully cover the gradient from small to large trees because all the studied trees were established in the 19th century.

Our research was not designed as a controlled experiment (sensu Jourez et al., 2001; Heinrich and Gärtner, 2008; Tumajer and Treml, 2019) and purely reference trees were absent in our study. On the other hand, the previous studies have generally focused fine time scales, so long-lasting effects resulting in height growth limitations have not been fully evaluated. With the analysis of long anatomical series, our results clearly show that conduit size limitation increases with tree size. Therefore, we expect that a comparison with a group of reference trees would show an even greater impact of the hillslope processes on the smaller trees.

## Conclusion

In this study, we used long-term anatomical series to evaluate the effect of long-term slope movements on tree anatomical structure and stem axial dimensions. The trees growing on a slope of average steepness were affected by permanent soil and regolith creep. The external pressure generated by moving soil mass disturbed the tree growth, with thickening trees gradually stabilizing their position and soils on slopes. This process of slope stabilization was followed by a gradual decrease of stem eccentricity with increasing stem diameter. However, the high range of the effect of indexed TRW at stem diameters from ∼5–30 cm suggests high susceptibility of those trees to the hillslope processes. Increased eccentricity forced the trees to produce reaction wood, composed of smaller vessels, and a large proportion of non-conductive elements, and thus lowered hydraulic conductivity. Despite the different responses in the anatomical structure of both the tree species (a lower effect on the decrease of vessel size), the effect of TRW variance was identical and resulted in a significant reduction in the proportion of conductive elements and an overall decrease of the hydraulic conductivity in the tree-rings. Moreover, this effect increased with the stem size.

The relationship between vessel size and stem diameter followed the Hagen-Poisseuille law up to ∼15 cm in both species, suggesting a linear relationship between stem length and stem diameter. In larger trees, a significant decrease in the coefficient of basipetal widening was observed. This was caused most likely by a higher allocation of biomass to the root system and increased radial growth. The limitations given by the long-term production of reaction wood led to limitations of height growth. We thus conclude that the penalties paid for the stability of a tree on a slope are a reduction in its height growth, a loss of aboveground biomass, and lowered carbon sequestration.

## Data Availability Statement

The data sets used and analysed in this study are available from the corresponding author on reasonable request.

## Author Contributions

JK designed the study, collected, and analyzed the tree ring data, and wrote the majority of manuscript. PŠ, PD, IV, and MK initiated the project, collected the soil data, and worked on the completion of the manuscript. PD specifically participated in the data analysis. MK was responsible for the TLS data. All authors contributed to the article and approved the submitted version.

## Conflict of Interest

The authors declare that the research was conducted in the absence of any commercial or financial relationships that could be construed as a potential conflict of interest.

## Publisher’s Note

All claims expressed in this article are solely those of the authors and do not necessarily represent those of their affiliated organizations, or those of the publisher, the editors and the reviewers. Any product that may be evaluated in this article, or claim that may be made by its manufacturer, is not guaranteed or endorsed by the publisher.
