# Supplementary figures and images for: Hillslope Processes Affect Vessel Lumen Area and Tree Dimensions
Source: Front Plant Sci. 2021 Dec 3;12:778802. doi: 10.3389/fpls.2021.778802 (PMC8678277; doi:10.3389/fpls.2021.778802)

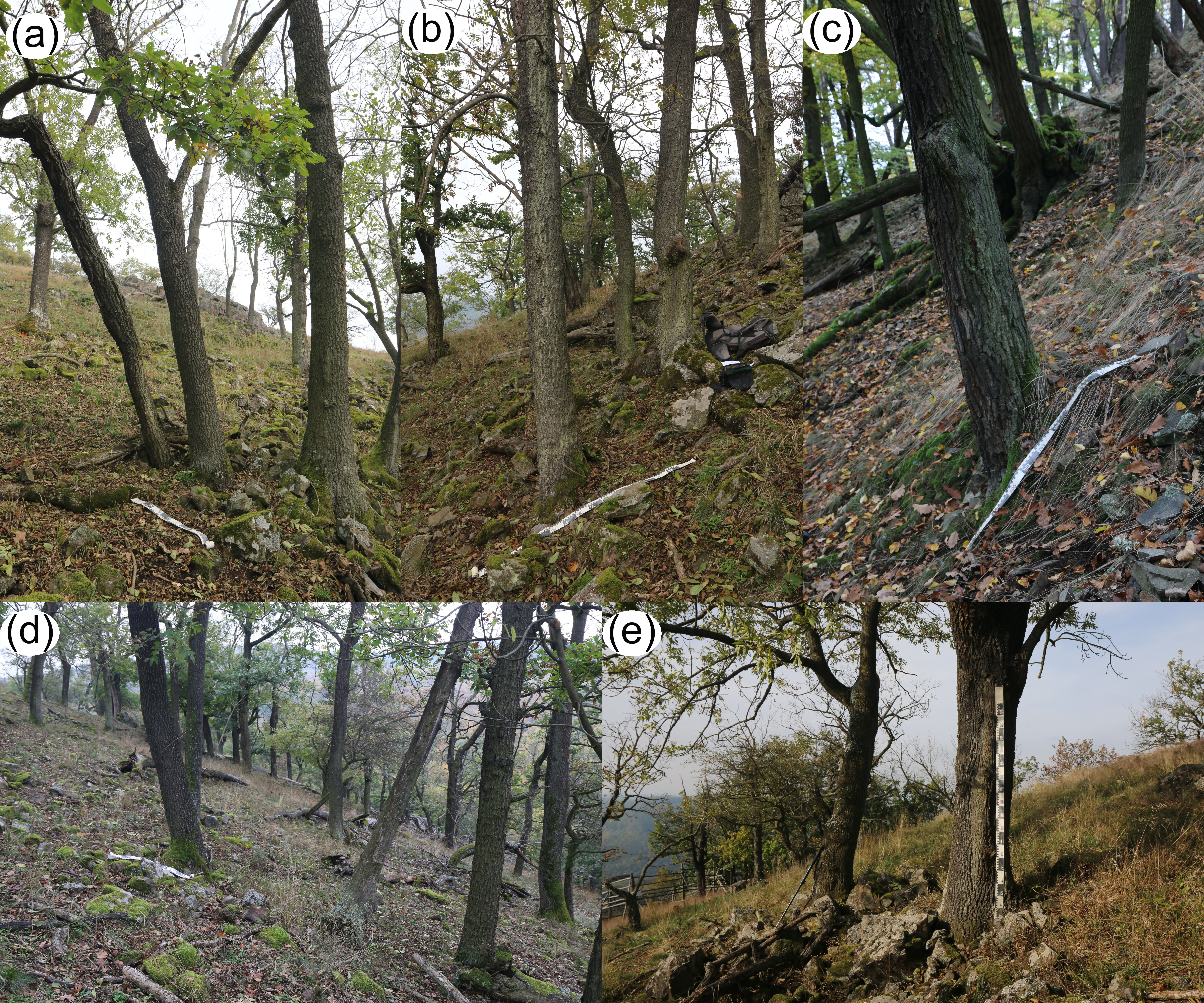

Supplement: Supplementary file 1 [file Image_1.JPEG]

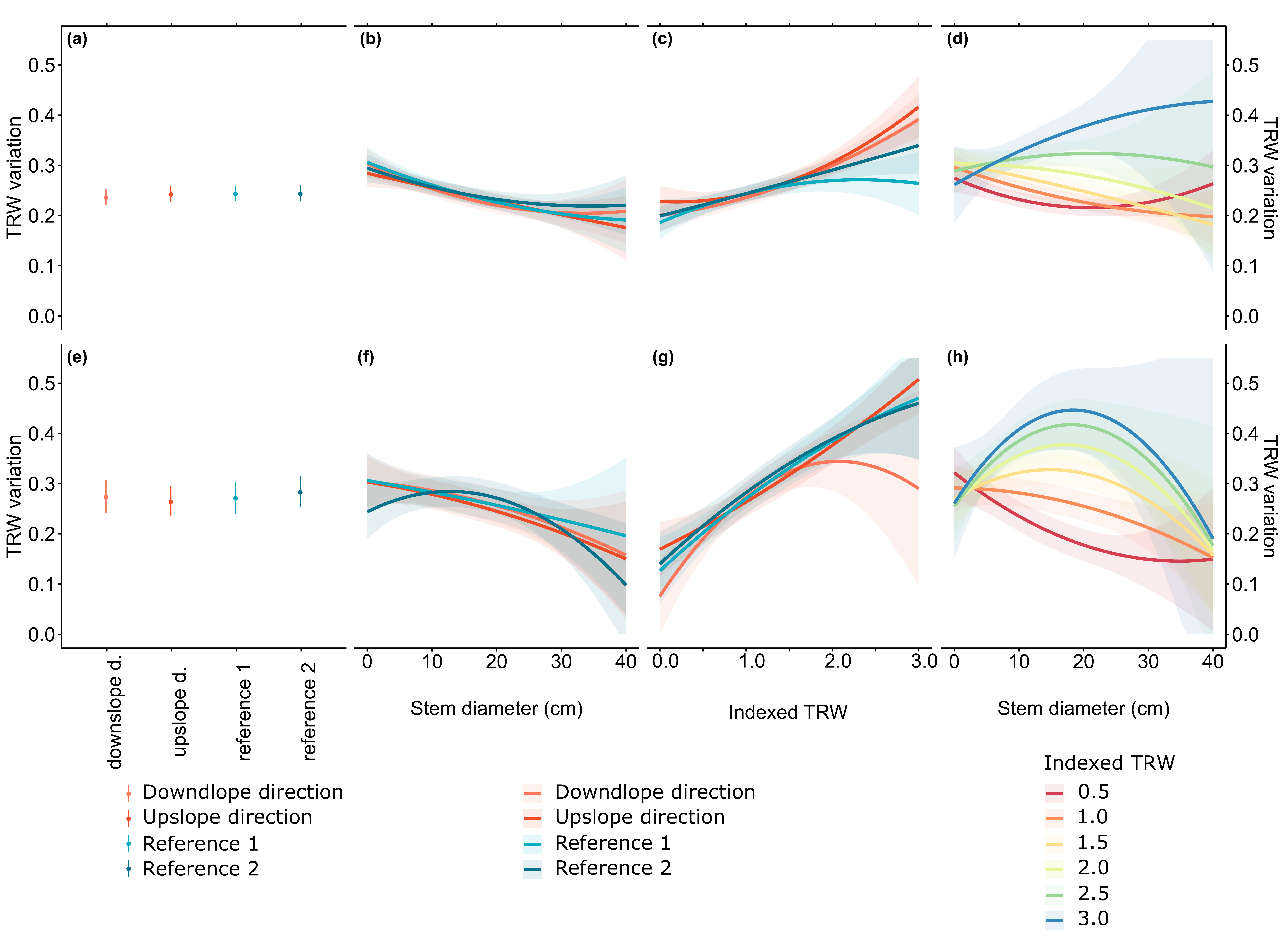

Supplement: Supplementary file 2 [file Image_2.JPEG]

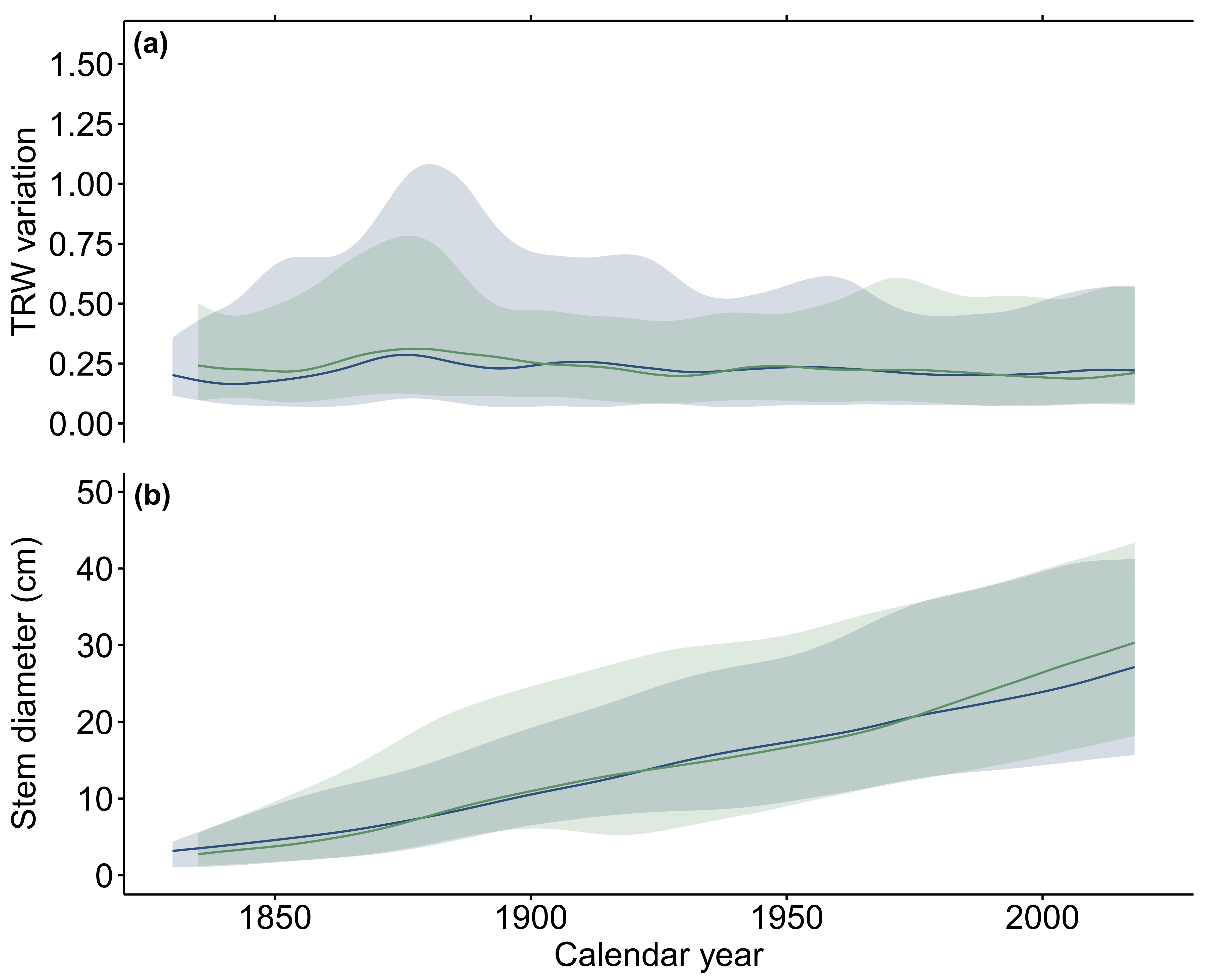

Supplement: Supplementary file 3 [file Image_3.JPEG]

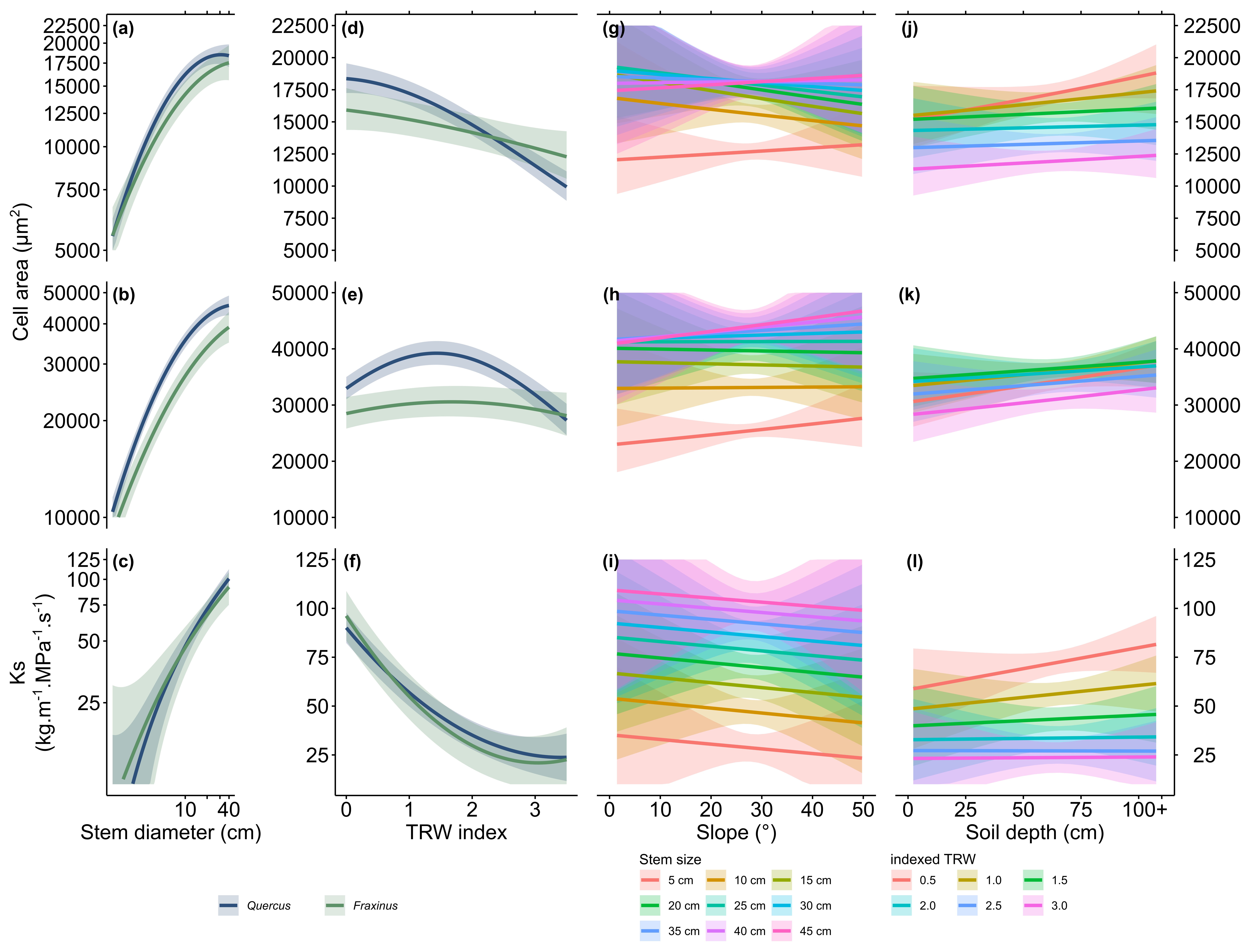

Supplement: Supplementary file 4 [file Image_4.JPEG]

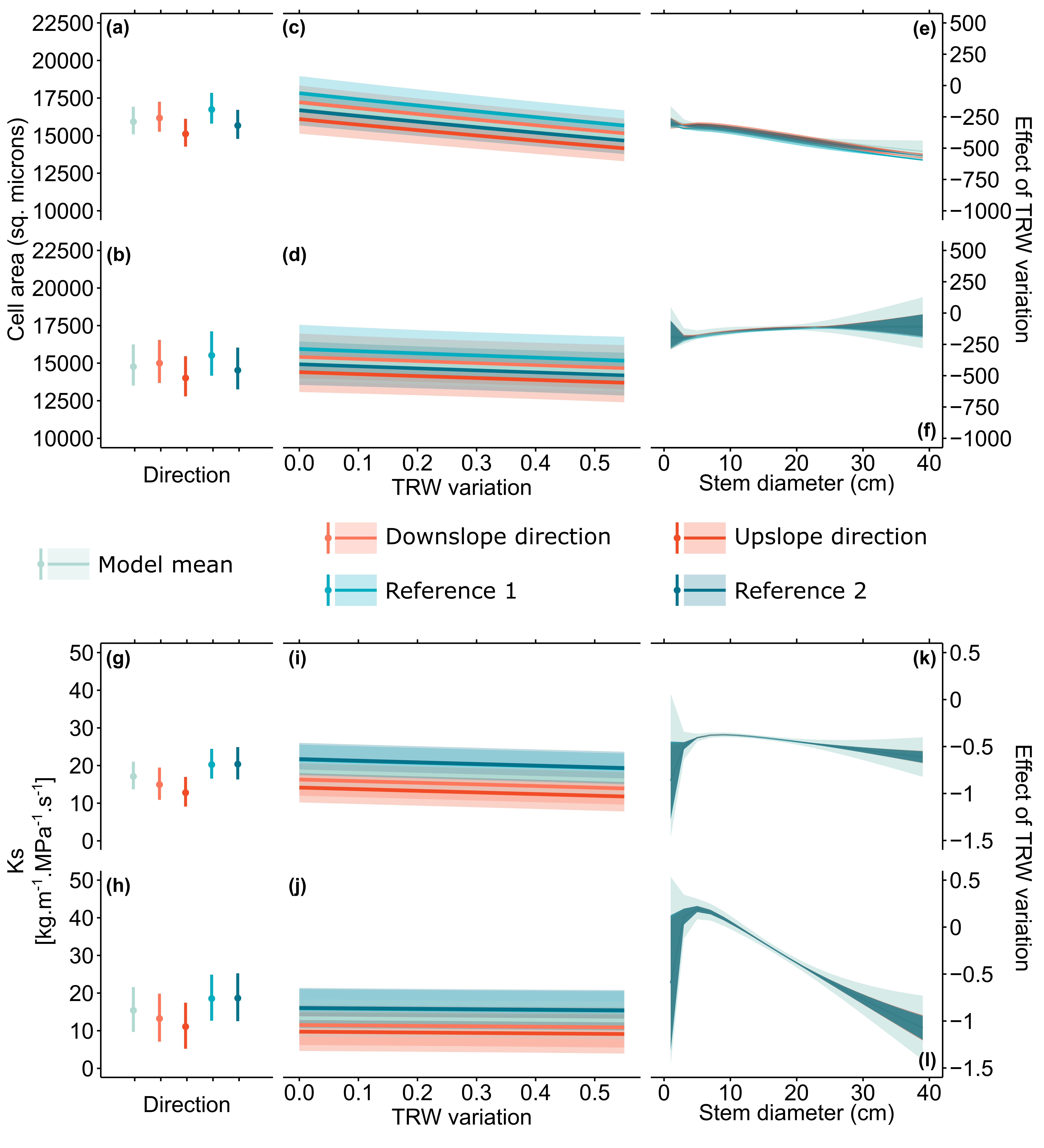

Supplement: Supplementary file 5 [file Image_5.JPEG]

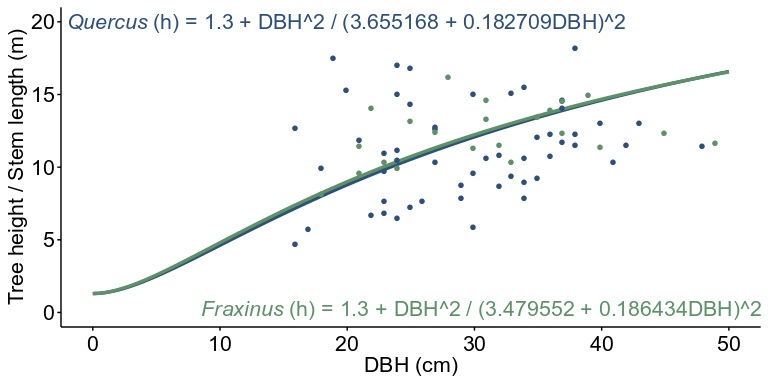

Supplement: Supplementary file 6 [file Image_6.JPEG]

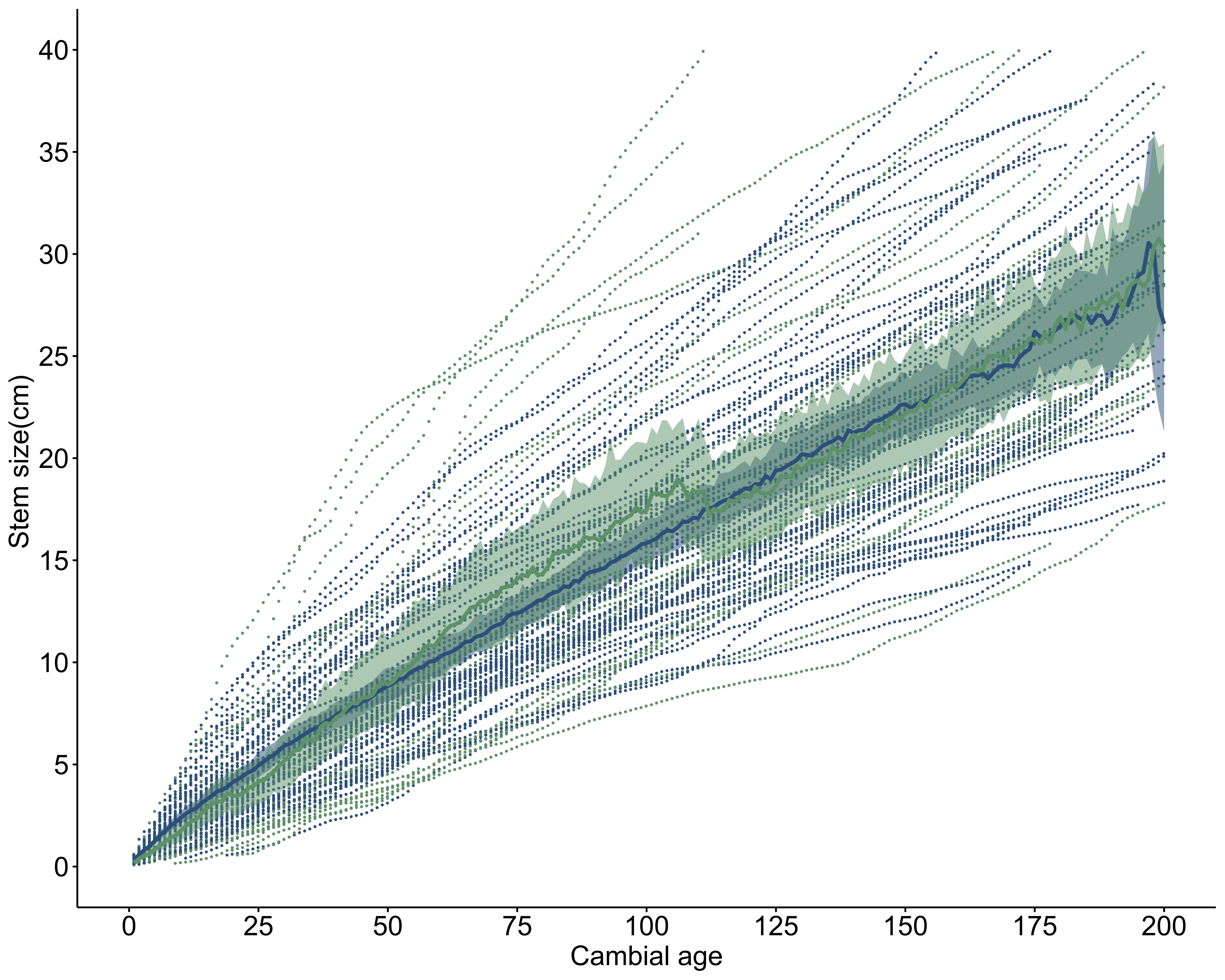

Supplement: Supplementary file 7 [file Image_7.JPEG]
