# Supplementary material for: Hillslope Processes Affect Vessel Lumen Area and Tree Dimensions
Source: Front Plant Sci. 2021 Dec 3;12:778802. doi: 10.3389/fpls.2021.778802 (PMC8678277; doi:10.3389/fpls.2021.778802)
Supplement: Supplementary file 8 [file Table_1.DOCX]

**Methods S1:** the R code of the model used for the simulation of TRW variance

lme(TRW.variation ~ Species+

Direction+

Soil.depth+

Slope+

poly(Stem.size,2)+

poly(TRW.index,2)+

Species:poly(TRW.index, 2)+

Species:poly(Stem.size,2)+

Direction:Soil.depth+

Direction:Slope+

Direction:poly(TRW.index, 2)+

Direction:poly(Stem.size, 2)+

Soil.depth:poly(TRW.index,2)+

Slope:Species+

Slope:poly(Stem.size, 2)+

Slope:poly(TRW.index, 2)+

poly(Stem size, 2):poly(TRW.index, 2)+

Species:poly(Stem.size, 2):poly(TRW.index, 2)+

Species:Direction:poly(Stem.size, 2)+

Species:Direction:+poly(TRW.index,2)+

Species:Slope:poly(TRW.index, 2)+

Direction:Slope:poly(TRW.index, 2)+

Direction:Soil.depth:poly(TRW.index, 2)+

Direction:poly(Stem.size, 2):poly(TRW.index, 2)+

Species:Direction:poly(Stem size, 2):poly(TRW.index, 2),

random= ~1|Tree.id,

correlation= corARMA(form= ~Calendar.year|Tree.id, p=3, q=2),

na.action=na.omit)
