# Supplementary material for: Hillslope Processes Affect Vessel Lumen Area and Tree Dimensions
Source: Front Plant Sci. 2021 Dec 3;12:778802. doi: 10.3389/fpls.2021.778802 (PMC8678277; doi:10.3389/fpls.2021.778802)
Supplement: Supplementary file 9 [file Table_2.DOCX]

**Methods S2:** the R code of the model used for the simulation of mean cell area (VLA_mean_), 90^th^ percentile of the mean vessel area (VLA_90_) and specific hydraulic conductivity (Ks).

lme(VLA_mean_/VLA_90_/Ks~ Species +

TRW.variation +

Slope +

Soil.depth +

poly(Stem.size, 2) +

poly(TRW.index, 2) +

Slope : Species +

TRW.variation : Species +

TRW.variation : poly(TRW.index, 2) +

poly(Stem.size, 2) : Species +

poly(Stem.size, 2) : Slope +

poly(Stem.size, 2) : TRW.variation +

poly(TRW.index, 2) : Slope +

poly(TRW.index, 2) : Soil.depth +

poly(TRW.index, 2) : Species +

poly(TRW.index, 2) : poly(Stem.size, 2) +

poly(TRW.index,2) : poly(Stem.size,2) : Species +

poly(TRW.index,2) : Slope : Species +

poly(Stem.size, 2) : TRW.variation: Species,

data= dataset, random= ~1|Tree.id,

correlation= corARMA(form=~Calendar.year|Tree.id, p=3, q=2),

na.action=na.omit)
