# Supplementary material for: Hillslope Processes Affect Vessel Lumen Area and Tree Dimensions
Source: Front Plant Sci. 2021 Dec 3;12:778802. doi: 10.3389/fpls.2021.778802 (PMC8678277; doi:10.3389/fpls.2021.778802)
Supplement: Supplementary file 10 [file Table_3.DOCX]

**Methods S3:** the R code of the model used for the simulation of mean cell area (VLA_mean_) and specific hydraulic conductivity (Ks) in cored directions

lme(VLAmean/Ks ~ Species +

Direction +

TRW.variation +

Slope +

Soil.depth +

poly(Stem.size,2) +

poly(TRW.index,2) +

Direction : poly(Stem.size,2)+

Direction : poly(TRW.index,2)+

TRW.variation : Species +

TRW.variation : poly(TRW.index,2) +

TRW.variation : poly(Stem.size,2)+

Slope : Species +

Slope : poly(Stem.size,2)+

Slope : poly(TRW.index,2)+

Soil.depth : poly(TRW.index,2) +

poly(Stem.size,2) : Species +

poly(Stem.size,2) : poly(TRW.index,2) +

poly(TRW.index,2) : Species +

TRW.variation : poly(Stem.size,2) : Species +

Slope : poly(TRW.index,2) : Species +

poly(Stem.size,2) : poly(TRW.index,2) : Species +

Direction : poly(Stem.size, 2): poly(TRW.index, 2),

data= dataset, random= ~1|Tree.id,

correlation= corARMA(form=~Calendar.year|Tree.id, p=3, q=2),

na.action=na.omit)
